# Severe Rhabdomyolysis in an Elderly Patient With Diabetes and Vascular Disease: Interplay of Statin Therapy, Sodium-Glucose Cotransporter-2 (SGLT2) Inhibition, and Thiazide-Induced Hypokalemia

**DOI:** 10.7759/cureus.95693

**Published:** 2025-10-29

**Authors:** Darpan Kothia, Bola Habeb, Felix Ermolenko

**Affiliations:** 1 Internal Medicine, Florida State University College of Medicine/ Ascension Sacred Heart, Pensacola, USA; 2 Internal medicine, Florida State University College of Medicine/ Ascension Sacred Heart, Pensacola, USA

**Keywords:** non-traumatic rhabdomyolysis, polypharmacy, sodium-glucose cotransporter-2 (sglt-2) inhibitors, statin-induced rhabdomyolysis, thiazide diuretics in elderly

## Abstract

Rhabdomyolysis is a potentially life-threatening complication of drug therapy, particularly in elderly patients exposed to polypharmacy. Statins are a well-known cause, while thiazide-induced hypokalemia and volume depletion can further predispose to muscle injury. Sodium-glucose cotransporter-2 (SGLT2) inhibitors such as empagliflozin provide cardio-renal benefits but may potentiate statin toxicity and exacerbate dehydration and electrolyte imbalance, amplifying rhabdomyolysis risk. We report a 78-year-old woman with diabetes, hypertension, hyperlipidemia, and carotid artery disease, hypothyroidism, who developed severe rhabdomyolysis, acute kidney injury, and transaminitis while taking simvastatin, hydrochlorothiazide, and empagliflozin. She presented with generalized weakness, muscle soreness, and exertional dyspnea; labs showed a creatine kinase of 12,165 U/L, hypokalemia of 2.9 mmol/L, and elevated troponin. Management included intravenous hydration, electrolyte repletion, and discontinuation of offending agents, resulting in clinical and biochemical improvement. This case underscores the multifactorial etiology of rhabdomyolysis in elderly vascular patients, where dehydration and hypokalemia from thiazides and SGLT2 inhibitors may synergize with statin-induced myotoxicity, complicating evaluation when troponin is elevated without ischemic changes. Clinicians should maintain a high index of suspicion for rhabdomyolysis in patients with new muscle pain, weakness, or fatigue after starting empagliflozin, especially when combined with statins and diuretics, and prioritize early recognition, medication review, and hydration to prevent serious complications.

## Introduction

Rhabdomyolysis is a clinical syndrome resulting from the rapid breakdown of striated muscle fibers, leading to the release of intracellular components- including creatine kinase (CK), myoglobin, potassium, phosphate, and uric acid - into the bloodstream [1]. The classic symptom triad consists of muscle pain, weakness, and dark-colored urine, due to myoglobinuria, although presentations are variable and up to half of patients may lack these hallmark features [1,2]. Additional symptoms can include muscle swelling, fever, fatigue, nausea, and vomiting, and some cases may be asymptomatic [2].

Epidemiologically, rhabdomyolysis is relatively rare but carries significant morbidity and mortality, primarily due to complications such as acute kidney injury (AKI), which develops in 13-50% of cases depending on etiology and clinical context [3]. The condition affects a broad demographic, with risk factors spanning both acquired and inherited domains. Acquired triggers include trauma, prolonged immobilization, strenuous exercise, extreme temperatures, infections, prescription and illicit drugs (notably statins and other lipid-lowering agents), alcohol, toxins, and metabolic or endocrine disturbances [1,3]. Inherited predispositions involve genetic defects in muscle metabolism, such as disorders of glycogen metabolism, fatty acid oxidation, mitochondrial dysfunction, and channelopathies, with RYR1 gene variants being a notable contributor [4].

Exertional rhabdomyolysis is particularly observed in athletes and military personnel exposed to intense or unaccustomed physical activity, especially under hot and humid conditions, but can also affect untrained individuals [5]. Other non-traumatic causes include viral infections (e.g., SARS-CoV-2), autoimmune myopathies, and electrolyte abnormalities [4,5]. The diagnosis is confirmed by a marked elevation in serum CK, typically five times above the upper limit of normal, and supported by laboratory findings of myoglobinuria and other muscle injury markers [1,4].

Statins are a cornerstone of cardiovascular risk reduction, but their use can rarely be complicated by myopathy and rhabdomyolysis [6]. Risk increases in elderly patients, those with renal impairment, and in the setting of drug-drug interactions. Simvastatin, a lipophilic statin, carries relatively higher myotoxic potential compared with hydrophilic agents [7].

Sodium-glucose cotransporter-2 (SGLT2) inhibitors, such as empagliflozin, improve cardiovascular and renal outcomes in patients with diabetes. However, case reports and pharmacokinetic studies suggest that these drugs may interact with hepatic transporters involved in statin metabolism, increasing systemic exposure and muscle toxicity [8-10]. Hydrochlorothiazide, commonly prescribed for hypertension, predisposes to hypokalemia, a well-recognized precipitant of rhabdomyolysis through muscle membrane destabilization [11].

We report a case of severe rhabdomyolysis in an elderly woman on simvastatin, empagliflozin, and hydrochlorothiazide, complicated by AKI and transaminitis. This case highlights the synergistic risk of polypharmacy and the clinical challenge of interpreting elevated troponin in rhabdomyolysis.

## Case presentation

A 78-year-old woman with a medical history of type 2 diabetes mellitus, hypertension, hyperlipidemia, hypothyroidism, bilateral carotid artery stenosis (status post bilateral endarterectomy 10 years ago after a transient ischemic attack), and chronic anemia secondary to gastrointestinal blood loss from internal hemorrhoids presented to the emergency department with five days of progressive generalized weakness and diffuse muscle soreness. She reported symptom onset five days prior to presentation, characterized by generalized muscle weakness, which was gradually progressive, leading to difficulty in taking stairs and to a point where she was unable to walk more than 1-2 minutes at ground level and making routine activities more effortful. She also noted occasional exertional shortness of breath and palpitations but denied trauma, strenuous activity, recent fever, headache, abdominal pain, burning micturition, vomiting or diarrhea, recent alcohol or recreational drug use, recent sick contacts, or immobilization. Her home blood pressure readings were around 150/90 mmHg, and she reported adherence to her medications.

Her home medications included empagliflozin 25 mg daily (initiated 4 months prior), hydrochlorothiazide 25 mg(since last 4 years) and simvastatin 40 mg daily, lisinopril 20mg, metformin 500 mg daily, clopidogrel 75 mg, levothyroxine 75 µg ( all since last 10 years), and oral iron and vitamin B12 supplements (for iron deficiency and B12 deficiency as per laboratory findings).

Clinical findings

On admission, she was afebrile with blood pressure 145/55 mmHg, heart rate 96 beats/minute, respiratory rate 17 breaths/min, and oxygen saturation 96% on room air. She appeared comfortable, with no focal neurologic deficits, and had strength of 5/5 in all four limbs; however, due to muscle soreness, particularly in proximal muscles, she had difficulty walking, leading to buckling of the lower limb after walking 1-2 minutes. Mild lower extremity edema was noted, but cardiopulmonary and abdominal examination were otherwise unremarkable.

Diagnostic assessment

The values below illustrate the course of creatinine kinase, electrolyte imbalance, and hepatic/renal function. Creatinine Kinase and transaminases peaked early and declined with hydration and medication withdrawal. Troponin remained elevated but stable, consistent with non-ischemic muscle injury. Urinalysis showed 3+ blood with no red blood cells, consistent with myoglobinuria.

Laboratory results obtained on admission are demonstrated in Table 1.

**Table 1:** Laboratory trends during hospitalization (Day 1–4). WBC: white blood cell; ALT: alanine aminotransferase; AST: aspartate aminotransferase; INR: international normalized ratio; BUN: blood urea nitrogen; TSH: thyroid-stimulating hormone *Abnormal laboratory findings

Parameter	Day 1	Day 2	Day 4	Reference Range
Hemoglobin (g/dL)	11.0*	10.7*	10.5*	12–16
WBC (×10³/µL)	9.6	7.7	7.2	4.0–11.0
Platelets (×10³/µL)	193	187	161	150–450
Creatinine kinase (U/L)	12,165*	5,994*	4,448	30–200
AST (U/L)	325*	181*	141*	10–40
ALT (U/L)	198*	142*	128*	7–56
Alkaline phosphatase (U/L)	99	-	-	40-150
Total bilirubin (mg/dL)	1.1	-	-	0.2-1.2
INR	1	-	-	-
Creatinine (mg/dL)	1.23*	0.99	0.82	0.6-1.1
BUN (mg/dL)	22*	18	18	7-20
Glomerular filtration rate (mL/min/1.73 m²)	42*	52*	62*	>90
Sodium (mmol/L)	140	136	138	135-145
Potassium (mmol/L)	2.9*	3.6	4.0	3.5-5.1
Corrected calcium levels (mg/dL)	9.0	8.6	8.5	8.5 - 10.2 mg/dL
Phosphorus levels (mg/dl)	6.8	7.2	5.1	2.5 - 4.5 mg/dL
Troponin (ng/mL)	0.332*	0.347*	0.297*	<0.01
Albumin (g/dL)	3.2*	-	-	3.5-5.0
TSH (µU/mL)	0.662	-	-	0.5 - 5
Blood glucose (mg/dL)	193*	-	-	70-99
HbA1C (%)	6.3	-	-	≤6.5
Urinalysis*	3+ Blood, 0 RBCs Glucose + Protein >30mg/dL	-	-	—

In view of the clinical presentation of weakness and muscle soreness, elevated transaminase and creatinine kinase, myoglobinuria, and AKI, a diagnosis of drug-induced rhabdomyolysis was made, and additional blood tests were done to rule out other causes of rhabdomyolysis (Table 2).

**Table 2:** Additional Laboratory during hospitalization. Hepatitis A IgM: hepatitis A virus Immunoglobulin M; Hepatitis Bs Ag: hepatitis B virus surface antigen; Hepatitis C Ab: hepatitis C virus antibody; Hepatitis B core IgM: hepatitis B virus core antibody, Immunoglobulin M; HIV Ag, Ab: human immunodeficiency virus antigen, antibody; LDL: low-density lipoprotein

Parameter	Results	Reference Range
Serum acetaminophen (mcg/mL)	<3.0	≤30
Serum salicylate (mg/dL)	<5.0	5–29
Ethanol (mg/dL)	<10	12–21
Hepatitis A IgM	Non-reactive	-
Hepatitis Bs Ag	Non-reactive	-
Hepatitis B core IgM	Non-reactive	-
Hepatitis C Ab	Non-reactive	-
HIV Ag, Ab combo screen	Non-reactive	-
Urine drug screen	Negative	Methamphetamine, Cocaine, Opiates, Phencyclidine, Methadone, Cannabinoids, Barbiturates, Benzodiazepines
Total cholesterol (mg/dL)	156	<200
LDL cholesterol (mg/dL)	73	Target <100

**Figure 1:**
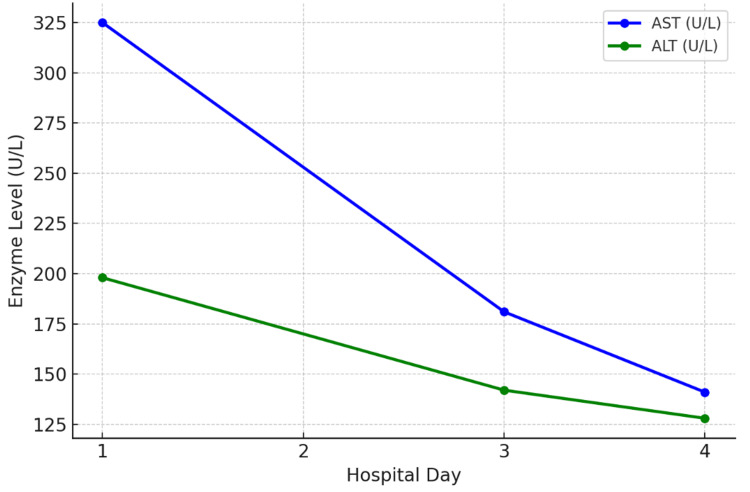
Transaminases trend during hospital stay ALT: alanine transaminase; AST: aspartate transaminase

**Figure 2:**
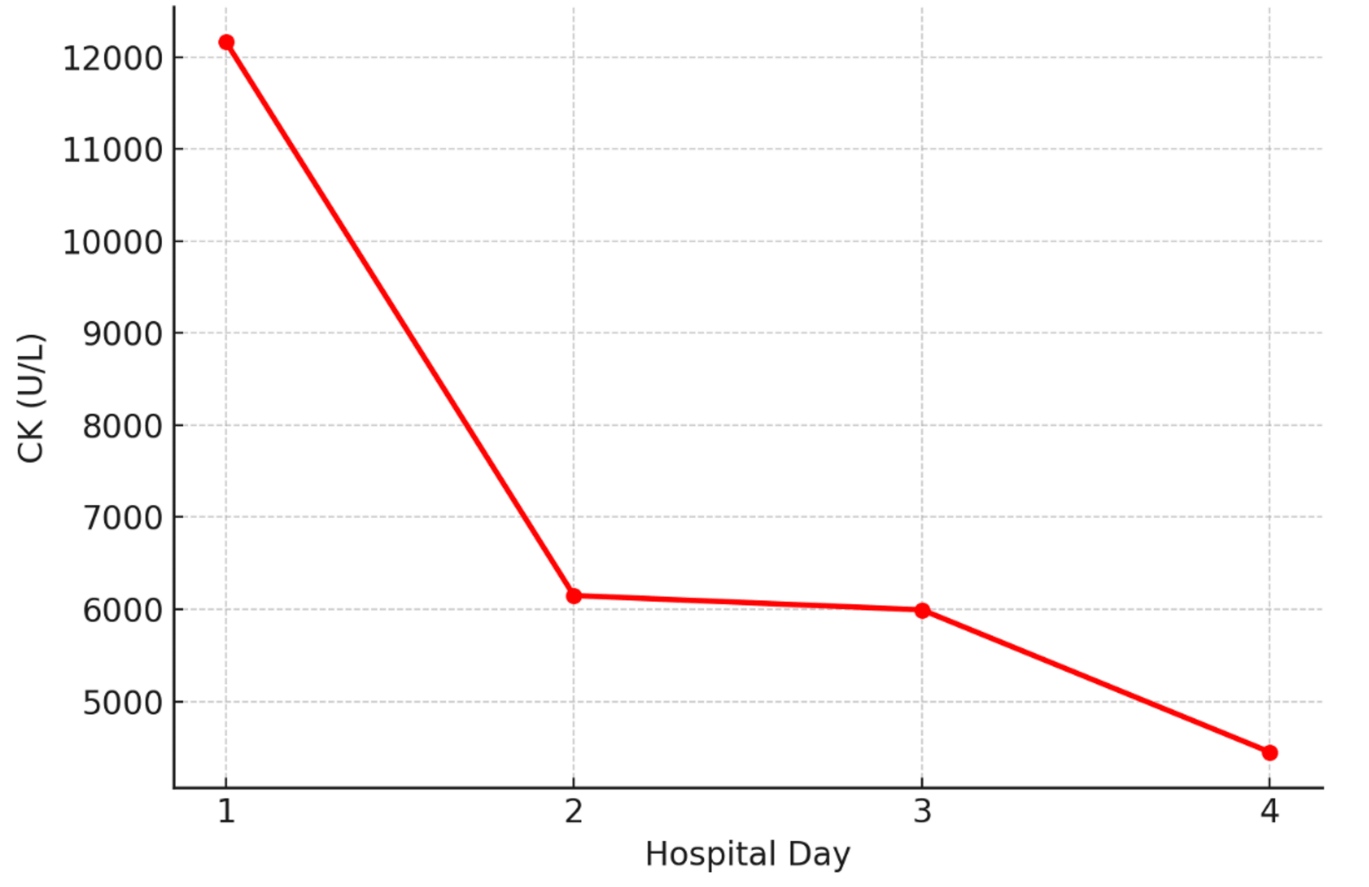
Total creatine kinase trend during hospital stay

The trend of AST, ALT, and creatinine kinase during admission and response to treatment has been demonstrated in Figures 1-2 in a graph pattern.

Imaging and cardiac evaluation

**Figure 3:**
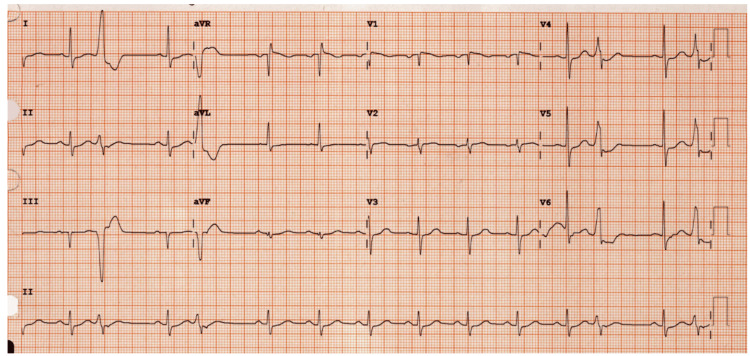
ECG with occasional premature ventricular contractions (PVCs)

The electrocardiogram showed occasional premature ventricular beats (PVCs) but no ischemic changes (Figure 3). Echocardiogram revealed normal right and left ventricular size and function (ejection fraction 65%) with mild-moderate calcific mitral stenosis. Chest X-ray and abdominal ultrasound showed no acute pathology.

Hospital course and management

Simvastatin, empagliflozin, and hydrochlorothiazide were discontinued on admission. The patient was clinically dehydrated on admission, with an elevated BUN/creatinine ratio supporting intravascular depletion. Management included IV isotonic fluid resuscitation targeting urine output ≥200 mL/hour, which was achieved consistently during hospitalization, along with gradual correction of hypokalemia using a total of 140 mEq of potassium supplementation over the course of hospitalization. Serial monitoring demonstrated a downward trend in CK and liver enzyme levels, accompanied by gradual improvement in her symptoms.

Cardiology was consulted, and inpatient ischemic evaluation was deferred, given the stability of troponin levels and absence of ischemic changes on electrocardiography. The patient was discharged in stable condition with recommendations for outpatient follow-up, including home physical therapy. She was discharged on metformin 500 mg once daily along with dietary modification, as her HbA1c was well controlled at 6.3% for her age. Outpatient follow-up was advised for reassessment of glycemic status and possible future adjustment of therapy. The patient declined a future trial of an alternative statin, such as rosuvastatin, due to adverse experience and preference to avoid statin rechallenge. For long-term lipid management, ezetimibe was advised as the next pharmacologic option, with future consideration of PCSK9 inhibitors (Proprotein convertase subtilisin/kexin type 9 inhibitors) should her LDL cholesterol remain above target levels despite therapy.

At the time of follow-up communication, no recurrence of symptoms or CK elevation was reported.

## Discussion

This case illustrates a rare but clinically significant triad of simvastatin-induced myopathy, thiazide-induced hypokalemia, and possible SGLT2 inhibitor potentiation through osmotic diuresis and intravascular volume depletion, which can impair muscle perfusion and augment statin-induced severe rhabdomyolysis. Simvastatin, a lipophilic statin, carries a higher myotoxic risk, especially in the setting of prolonged exposure [8-10]. The concurrent use of all these medications created a setting in which multiple pharmacologic mechanisms converged to precipitate severe rhabdomyolysis. Polypharmacy in such patients can lead to synergistic toxicities. Clinicians should remain vigilant for muscular symptoms in patients recently initiated on SGLT2 inhibitors who are already receiving statins and diuretics.

Hydrochlorothiazide-induced hypokalemia likely served as a critical cofactor. Hypokalemia destabilizes skeletal muscle membranes and increases susceptibility to muscle injury [11,12]. When combined with statin toxicity and impaired drug clearance, it likely lowered the threshold for rhabdomyolysis.

Experimental studies suggest that SGLT2 inhibitors may impact statin pharmacokinetics via transporter interactions. While healthy volunteer trials of empagliflozin with simvastatin showed only modest changes in statin exposure and no adjustment was deemed necessary [13], it is well-established in pharmacokinetic literature that statins are substrates of OATP1B1 (Organic Anion Transporting Polypeptide 1B1), CYP3A4 pathway and P-gp (P-glycoprotein), and that inhibition of these transporters can lead to increased statin systemic concentrations [14].

Similar cases have been reported individually in the literature (Table 3); however, the convergence of all three pharmacologic triggers-statin therapy, SGLT2 inhibition, and thiazide-induced hypokalemia - in a single patient with vascular disease. This underscores the novelty and educational importance of the present case. The absence of trauma, exertion, infection, or alcohol use further supports a drug-induced etiology.

**Table 3:** Comparison of Published Cases with the Present Case HCTZ: Hydrochlorothiazide The current case presented with higher CK levels and concurrent mild troponin elevation compared to previously published cases, yet showed rapid biochemical and clinical recovery after drug discontinuation and hydration. This distinction underscores the multifactorial nature and reversibility of the interaction when promptly recognized.

Mechanism/Trigger	Age & Sex	Published Report(s)	Key Findings	Overlap With Present Case
Statin-associated rhabdomyolysis in the elderly	67 years, Male	Ezad et al., 2018 [6]	Elderly patient on simvastatin developed severe rhabdomyolysis	Present patient was elderly and on simvastatin
SGLT2 inhibitor–statin interaction	57 years, Male	Udayashankar et al., 2025 [9]	Rhabdomyolysis due to SGLT2–statin pharmacokinetic interaction	Patient on empagliflozin + simvastatin
Thiazide-induced hypokalemic rhabdomyolysis	72 years, Female	Antoniadis et al., 2003 [11]	HCTZ-induced hypokalemia (K⁺ 2.9 mmol/L) leading to rhabdomyolysis	Patient on hydrochlorothiazide developed hypokalemia
Thiazide as precipitant of statin toxicity	68 years, Male	He et al. 2018 [12]	Thiazide-induced hypokalemia triggered statin-related rhabdomyolysis	HCTZ + statin contributed synergistically

To further assess the likelihood that this event was drug-induced, the Naranjo Adverse Drug Reaction Probability Scale was applied [15]. The total score of 7 indicates a probable relationship between the combination therapy and rhabdomyolysis. This supports our conclusion that the interaction among simvastatin, hydrochlorothiazide, and empagliflozin likely contributed to the patient’s presentation (Table 4).

**Table 4:** Naranjo Adverse Drug Reaction Probability Scale Naranjo Adverse Drug Reaction Probability Scale — A validated tool used to assess the likelihood that a drug caused an observed adverse event. Scoring interpretation: ≥9 = Definite, 5–8 = Probable, 1–4 = Possible, 0 = Doubtful

No.	Question/Criterion	Response	Score	Explanation
1	Previous reports on this reaction	Yes	+1	Statin-induced rhabdomyolysis is well documented, particularly with concurrent thiazide or SGLT2 inhibitor use.
2	Adverse event appeared after the suspected drug was administered	Yes	+2	Empagliflozin was initiated 4 months prior to symptom onset.
3	Improvement on discontinuation (dechallenge)	Yes	+1	Marked improvement in symptoms and CK after withdrawal of simvastatin, hydrochlorothiazide, and empagliflozin.
4	Reappearance on readministration	Not done	0	The patient declined rechallenge.
5	Alternative causes reasonably excluded	Yes	+2	No trauma, infection, hypothyroidism exacerbation, or other metabolic/toxic causes identified.
6	Reappearance with placebo	Not applicable	0	—
7	Drug detected in toxic concentrations	Not tested	0	—
8	Dose–response relationship	Not applicable	0	Stable doses before onset.
9	Similar reaction to same or similar drugs previously	No	0	—
10	Objective confirmation of reaction	Yes	+1	Elevated CK and liver enzymes with improvement after drug withdrawal.

The markedly elevated troponin level initially raised concern for myocardial ischemia. However, the absence of ischemic symptoms, dynamic ECG changes, or echocardiographic abnormalities supported a non-ischemic etiology. Troponin elevation in rhabdomyolysis is well described and may result from skeletal muscle release or impaired renal clearance, particularly in acute kidney injury [16]. Similarly, the observed transaminitis with AST predominance was more consistent with muscle injury than primary hepatocellular damage [17]. These findings emphasize an important diagnostic pitfall: troponin and transaminase elevations in rhabdomyolysis can mimic acute coronary or hepatic injury, potentially leading to unnecessary interventions. Early recognition of this syndrome and timely interventions-including prompt withdrawal of offending agents, aggressive intravenous hydration, and gradual electrolyte repletion-remain the cornerstone of successful management.

The American College of Cardiology and the National Lipid Association recommend that, following resolution of rhabdomyolysis and normalization of CK levels, rechallenge with a different statin-such as rosuvastatin, which is less lipophilic and has a distinct pharmacokinetic profile-may be considered, starting at the lowest possible dose (e.g., 5 mg daily) and titrating as tolerated, with ongoing monitoring for muscle symptoms and CK elevations [18-19]. Rosuvastatin is preferred in patients with prior adverse reactions to lipophilic statins due to its reduced risk of drug-drug interactions and lower tissue penetration [19]. This patient had been on simvastatin 40 mg daily prescribed by her primary care physician for approximately 10 years following carotid endarterectomy, with good tolerance and well-controlled LDL levels. Given her stability on this regimen, it is presumed that her physician continued the same medication and dosage.

If muscle symptoms or CK elevations recur, statin therapy should be discontinued and alternative lipid-lowering strategies considered, such as ezetimibe or PCSK9 inhibitors [20].

Limitations of the case report: Muscle biopsy or genetic myopathy testing was not performed, as the patient’s clinical improvement and rapid CK decline following drug discontinuation and hydration supported a toxic-metabolic etiology, making primary myopathy less likely. We acknowledge the absence of statin plasma concentration measurements and pharmacogenetic testing (e.g., SLCO1B1 variants) as study limitations. The synergistic mechanism between empagliflozin and statin toxicity remains hypothesized rather than definitively proven. The proposed pathophysiologic link is supported by existing case reports and pharmacologic plausibility but has not yet been confirmed through controlled studies. We concur that pharmacovigilance data and larger, prospective studies are required to confirm the observed interaction and clarify its incidence and mechanisms.

## Conclusions

This case highlights the complexity of medication management in elderly patients with multiple comorbidities. The combination of simvastatin, empagliflozin, and hydrochlorothiazide produced a synergistic toxicity resulting in rhabdomyolysis, AKI, transaminitis, and marked troponin elevation.

Preventive strategies should include baseline CK and electrolyte assessment before initiating combination therapy, periodic renal function monitoring, and routine medication review to identify overlapping risk factors for myopathy. Early recognition, prompt withdrawal of the offending drugs, aggressive intravenous hydration, and gradual electrolyte correction were critical to the patient’s recovery. Clinicians should maintain vigilance for drug-drug interactions and consider rhabdomyolysis in elderly patients presenting with nonspecific weakness or myalgias. Importantly, elevations of troponin and transaminases in this context should be interpreted cautiously, as they may reflect systemic muscle injury rather than primary cardiac or hepatic pathology.

This case adds to the growing literature on polypharmacy-induced rhabdomyolysis and serves as a reminder of the delicate balance required in cardiovascular risk management for elderly patients.
